# Effects of GnRH vaccination in wild and captive African Elephant bulls (*Loxodonta africana*) on reproductive organs and semen quality

**DOI:** 10.1371/journal.pone.0178270

**Published:** 2017-09-15

**Authors:** Imke Lueders, Debbie Young, Liana Maree, Gerhard van der Horst, Ilse Luther, Stephan Botha, Brendan Tindall, Geoffrey Fosgate, André Ganswindt, Henk J. Bertschinger

**Affiliations:** 1 GEOlifes-Animal Fertility and Reproductive Research, Frohmestr. 7, Hamburg, Germany; 2 Endocrine Research Laboratory, Department of Anatomy and Physiology, Faculty of Veterinary Science, University of Pretoria, Onderstepoort, South Africa; 3 African Elephant Research Unit, Plettenberg Bay, South Africa; 4 Department of Medical Biosciences, University of the Western Cape, Bellville, South Africa; 5 Department of Research and Scientific Services, National Zoological Gardens of South Africa, Pretoria, South Africa; 6 African Lion Safari, Cambridge, ON, Canada; 7 Robberg Veterinary Clinic, 56 Longships, Plettenberg Bay, South Africa; 8 Department of Production Animal Studies, Faculty of Veterinary Science, University of Pretoria, Onderstepoort, South Africa; Centre for Cellular and Molecular Biology, INDIA

## Abstract

**Objectives:**

Although the African elephant (*Loxodonta africana*) is classified as endangered by the International Union for Conservation of Nature (IUCN), in some isolated habitats in southern Africa, contraception is of major interest due to local overpopulation. GnRH vaccination has been promoted as a non-invasive contraceptive measure for population management of overabundant wildlife. We tested the efficacy of this treatment for fertility control in elephant bulls.

**Methods:**

In total, 17 male African elephants that were treated with a GnRH vaccine were examined in two groups. In the prospective study group 1 (n = 11 bulls, ages: 8–36 years), semen quality, the testes, seminal vesicles, ampullae and prostate, which were all measured by means of transrectal ultrasound, and faecal androgen metabolite concentrations were monitored over a three-year period. Each bull in the prospective study received 5 ml of Improvac^®^ (1000 μg GnRH conjugate) intramuscularly after the first examination, followed by a booster six weeks later and thereafter every 5–7 months. In a retrospective study group (group 2, n = 6, ages: 19–33 years), one examination was performed on bulls which had been treated with GnRH vaccine for 5–11 years.

**Results:**

In all bulls of group 1, testicular and accessory sex gland sizes decreased significantly after the third vaccination. In six males examined prior to vaccination and again after more than five vaccinations, the testis size was reduced by 57.5%. Mean testicular height and length decreased from 13.3 ± 2.6 cm x 15.2 ± 2.8 cm at the beginning to 7.6 ± 2.1 cm x 10.2 ± 1.8 cm at the end of the study. Post pubertal bulls (>9 years, n = 6) examined prior to vaccination produced ejaculates with viable spermatozoa (volume: 8–175 ml, sperm concentration: 410-4000x10^6^/ml, total motility: 0–90%), while after 5–8 injections, only 50% of these bulls produced ejaculates with a small number of immotile spermatozoa. The ejaculates of group 2 bulls (vaccinated >8 times) were devoid of spermatozoa. Faecal androgen metabolite concentrations measured in captive males decreased significantly after the fourth vaccination. None of the males entered musth during the treatment period.

**Conclusions:**

Our results showed a marked decrease in semen quality, testicle and secondary sex gland sizes following repeated GnRH vaccinations. After 2–4 years of continuous treatment every 5–7 months, the effects were similar to surgical castration.

## Introduction

Gonadotrophin releasing hormone (GnRH) is synthesized in specialized neuroendocrine cells in the hypothalamus and transported to the anterior pituitary via the hypothalamic-pituitary portal system, where it induces synthesis and release of the gonadotrophic hormones, follicle stimulating hormone (FSH) and luteinizing hormone (LH) [1]. Both gonadotrophic hormones are important for the regulation of male and female gonadal function (gametogenesis and steroidogenesis). In males, this includes the production of spermatozoa and testosterone in the testes. The concept of GnRH vaccines is based upon the interruption of the hypothalamic-pituitary-gonadal (HPG) axis by stimulating anti-GnRH antibody production. These antibodies neutralize endogenous GnRH and, subsequently, disrupt the downstream release of gonadotrophic hormones. Being able to target GnRH in order to suppress testicular function, without hormonal or surgical intervention, appears to be an elegant method of contraception. This is often referred to as down-regulation of testicular function in the peer-reviewed literature. Efficacy is, however, dependent on the achieved humoral immunity and may vary between and within species as well as GnRH vaccine formulations [2]. For instance, it has been shown that older stallions are more difficult to supress than younger ones [3,4]. While male rats show a strong response with almost a 100% arrest in spermatogenesis, in male dogs a decrease of only 5% in spermatogenic activity was reported [5].

Commercial GnRH vaccines have been designed for domestic animals and tested in both sexes of pigs [6], cattle [7] and horses [4,8]. GnRH vaccines have been suggested as means of pest or feral animal control, and have been tested in several wildlife species, such as white-tailed deer (*Odocoileus virginianus*, [9]), bison (*Bison bison*, [10]) and wild boars (*Sus scrofa*, [11]) with moderate to good success. Recent studies indicated that GnRH vaccines could be useful for the management of androgen driven behaviours in elephants and the hormone mediated musth condition seen in adult African (*Loxodonta africana*) and Asian elephant (*Elephas maximus*) bulls [12–14]. The GnRH vaccine Improvac® (Zoetis Animal Health, South Africa) has been frequently used as an extra-label drug in southern Africa to control androgen-related aggressive behaviour and musth in approximately 45 bulls over the past 10 years [13]. Independent case reports on a male [15] and a female Asian elephant [16] suggest that GnRH vaccines are capable of suppressing gonadal function after multiple monthly injections. After two to three treatments, a decrease in serum testosterone and faecal androgen metabolite concentration was found in male Asian [17] and African elephants [14], respectively. This was accompanied by behavioural improvement and/or attenuation or prevention of musth. A rise in anti-GnRH antibody titres could be confirmed after 3–5 monthly boosters in one female [16] and after 2–4 monthly boosters in six male Asian elephants [17]. Only one of the above studies investigated the effects of GnRH vaccines on the reproductive organs in a single Asian elephant [15] and none monitored the effect on semen quality in either species.

Although African elephant populations are under pressure, in southern Africa fertility control is warranted as capacities in smaller, fenced game reserves are exceeded. Due to the intra-abdominal location of the testes, surgical castration is impractical and expensive in these large animals, especially when conducted in the wild. Laparoscopic vasectomy has also been performed, but requires specialized operators and expensive equipment and is not without risks for the animal [18]. Vasectomy leads to irreversible infertility in elephant bulls, while androgen-driven behaviours remain unaffected. The aim of this study was to test the effects of the commercially available GnRH vaccine Improvac® (Zoetis, South Africa) on the HPG-axis in elephant bulls. Thus, we hypothesise a castration-like effect to occur on the internal reproductive organs, semen quality and faecal androgen metabolite concentrations in adolescent and mature African elephant bulls.

## Materials and methods

### Study animals

Captive (n = 13) and wild (n = 4) African elephant bulls were used for the study and examined between March 2011 and April 2014 (Table 1). In the prospective group 1, consisting of nine captive and two wild elephants, the effects of the GnRH vaccine were studied from prior to or soon after the primary vaccination, and thereafter every 5 to 12 months for a period of 3 years. Seven (five captive, two wild) of these bulls were naïve at the time of the first examination. The first follow-up examination took place either after primary and first booster immunizations (n = 5) or after three injections (n = 6, primary, first booster after 6 weeks and second booster five months later, Table 1). The animals in group 2, consisting of four captive and two wild bulls that had previously been treated with the GnRH vaccine in the same manner for 5–11 years (primary immunisation, first booster after 4–6 weeks followed by boosters every 5–7 months until the date of examination), were examined once only to assess long-term effects of the vaccine on semen quality and the reproductive organs. Age, shoulder height, calculated weight, treatment status and number of vaccinations and examinations for all bulls are provided in Table 1. Body weights were calculated as a mean of two previously published formulas on weight/height relationship of African elephant bulls [19,20]. This study was carried out in strict accordance with recommendations in the Guide for the Care and Use of Laboratory Animals of the National Institutes of Health. The research proposal was approved by the Animal Use and Care Committee of the University of Pretoria (Certificate # V016-12).

**Table 1:** Overview and details of all elephants treated and examined during the course of the study.

Elephant number	Wild or captive	Number of examinations	Study type	Age range^a^ (years)	Shoulder height^1^ (cm)	Calculated body weight^1^ (kg)	Number of GnRH vaccinations at first examination	Total number of GnRH vaccinations at last examination
1	captive	4	group 1	24–27	293–296	4172–4336	2	7
2	captive	4	group 1	24–27	276–287	3595–4025	2	7
3	captive	4	group 1	17–20	240–265	2401–3197	2	7
4	captive	4	group 1	12–15	230–256	2124–3232	2	7
5	captive	3	group 1	8–12	210–212	1566–1657	0	4
6	captive	4	group 1	10–13	225–237	1994–2316	0	8
7	captive	4	group 1	23–26	290–308	4147–4935	0	8
8	captive	4	group 1	23–26	292–310	4230–5029	0	8
9	captive	4	group 1	23–26	297–306	4443–4843	0	8
10	wild	4	group 1	30–33	302	4396	0	5
11	wild	4	group 1	15–18	247	2483	0	5
12	captive	1	group 2	26	293	4034	>5	>8
13	captive	1	group 2	19	248	2662	>5	>8
14	wild	1	group 2	28	317	5046	>8^b^	>8^b^
15	wild	1	group 2	33	336	5815	>8^b^	>8^b^
16	captive	1	group 2	20	263	3033	>8^b^	>8^b^
17	captive	1	group 2	30	336	5815	>8^b^	>8^b^

Group 1: multiple examinations over time; Group 2: single examination of long-term treated elephants

^a^ elephants were examined over a period of two to three years, therefore age, shoulder height and body weight are given as ranges as they increased over the course of the study

^b^ exact number of vaccinations for these animals is not known, but each bull had more than 8 injections at the time of examination

### GnRH vaccinations

The treatment protocol for both groups consisted of a primary immunization, followed by a first booster after 6 weeks, and thereafter boosters every 5–7 months. In group 1, animals were treated over the examination period of two to three years, in group 2 for 5–11 years. The vaccine was administered by deep intramuscular injection into the muscle mass of the hind leg. In captive elephants, this was mostly delivered by hand injection and occasionally with a dart gun. Wild bulls were injected by hand under anaesthesia (first injection) or remotely with a dart gun from a helicopter, in which case the gluteal muscle mass was targeted. Each immunization consisted of 5 ml Improvac® (Zoetis Animal Health, South Africa) containing 1000 μg GnRH-protein conjugate.

### Anaesthesia

In all but one captive bull procedures were carried out under standing sedation with medetomidine and butorphanol, as previously described [21]. One captive and all four wild bulls were fully immobilized using a combination of 12–15 mg etorphine (M99, Novartis Animal Health, Isando, South Africa) and 10–20 mg detomidine (Domosedan, Zoetis Animal Health, South Africa), South Africa) according to estimated weight and delivered remotely from a dart gun fired from a helicopter (wild bulls) or the ground (captive bull). Reversals were achieved with 100–150 mg diprenorphine (M5050, Novartis Animal Health, Isando, South Africa) and atipamezole HCL (Antisedan, Zoetis Animal Health, South Africa) administered intravenously.

### Ultrasound examination

**Fig 1:**
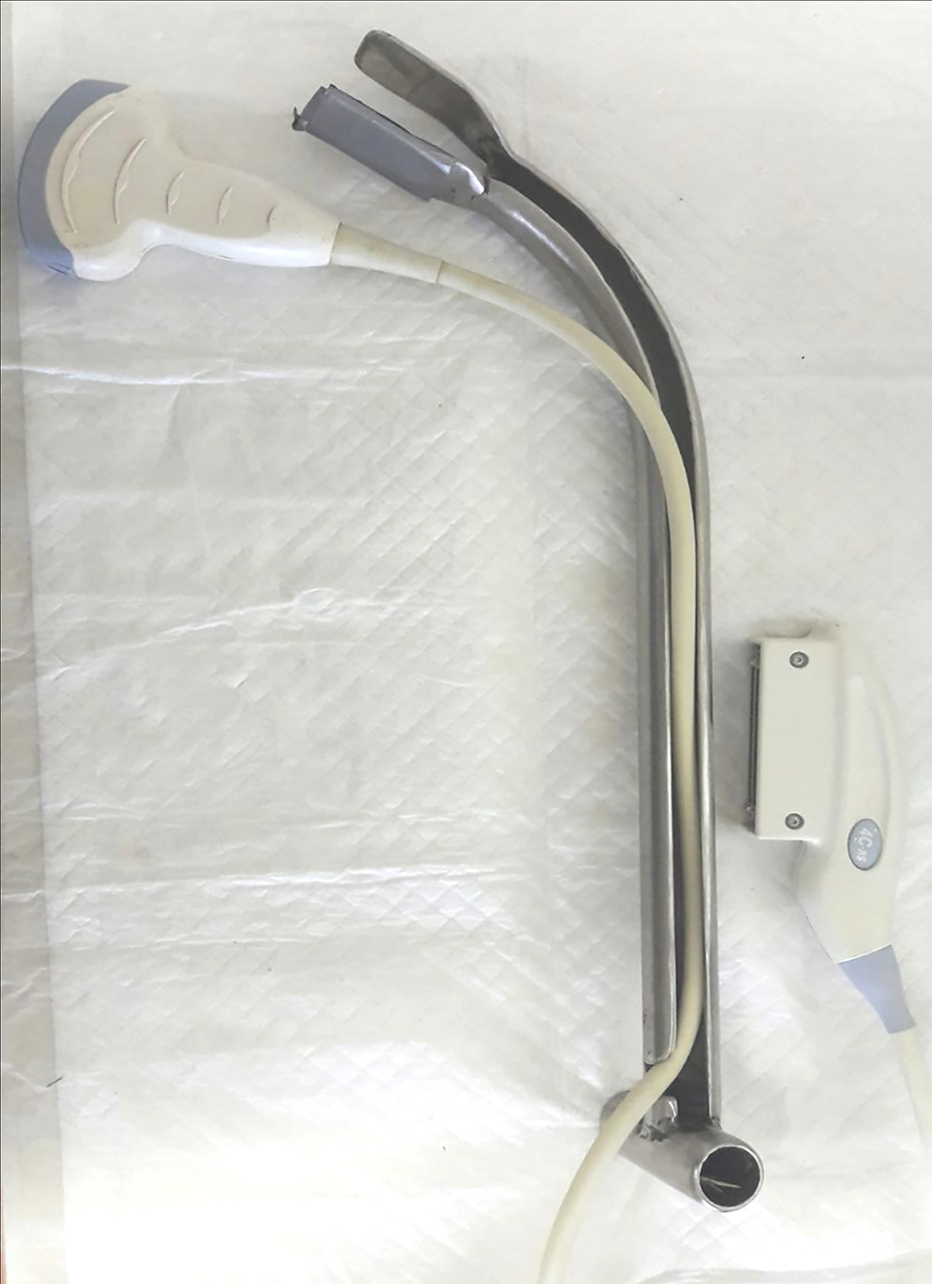
Convex ultrasound probe and steel extension used for transrectal examination of intra-abdominally situated testicles in elephant bulls.

Prior to ultrasound examination and semen collection of standing or recumbent bulls, faeces were removed manually after which the rectum was flushed with water using a hosepipe. Trans-rectal ultrasound examinations were performed as previously described [22,23]. A portable, battery-driven ultrasound machine (Logic e, General Electric (GE) Healthcare GmbH, 42655 Solingen, Germany) equipped with a 2–7 MHz convex probe was used. In elephants up to 11 years of age, the testes, which are located caudal to the kidneys, could be visualized using a hand-held ultrasound probe. In older bulls, an extension handle was necessary to reach the testes. The extension handle consisted of a 42 cm long hollow steel pipe with a T-shaped handle and a groove at the other end to hold the ultrasound probe, which was held in place with duct tape. The probe end of the extension was angled at 45° to facilitate the location of each testis (Fig 1).

Six to ten second long ultrasound video recordings, each of the ampullae, prostate, seminal vesicles and testes, were made for retrospective analysis. Frozen images were used to measure the length and height of each organ using the largest dimensions in each case. The diameter was calculated as a mean of both values for statistical analyses. Since accessory sex glands are mostly asymmetrical or amorphous-shaped organs, the circumference was also measured by circumscribing the organ. The outlined 2-D images were then used to calculate the respective areas for statistical analyses. Since testes and accessory sex glands are paired organs, the means of the combined measurements were calculated.

### Semen collection

Two semen collection methods were initially used and then consistently applied in subsequent examinations of individual bulls. Semen collection of bulls under standing sedation was performed by trans-rectal, manual massage of the prostate and ampullae of the vas deferentia as previously described for trained [24] or sedated Asian elephants [25]. The position of the ampullae and prostate was located by means of ultrasound. Once semen started dripping from the penis, it was collected in 50 ml polypropylene tubes (Greiner centrifuge tubes, Greiner Bio-One International GmbH, 4550 Kremsmünster, Austria) attached to funnels made from plastic rectal sleeves. The tubes were changed regularly to collect ejaculatory fractions and to avoid possible urine contamination of semen samples.

The method used for electro-ejaculation of one captive and four wild immobilised bulls was similar to the one described by Howard *et al*. [26]. A portable, battery driven cattle electro-ejaculator (El Toro 3, Electronic Research Group, Midrand, Johannesburg, South Africa) equipped with a hand-held probe, specifically designed for elephants, was used. The probe which was 15 cm in diameter was fitted with three ventral electrodes, each 20 cm by 3 cm and spaced 3 cm apart. The probe was placed into the rectum dorsal to the urethra at the depth of the ampullae and prostate. Low voltage stimuli were applied and gradually increased as described previously [27]. In our case, however, much lower voltages, not exceeding 16 V, resulted in ejaculation. The number and duration of stimuli was adapted according to the response of each male (urethral contractions, erection, mushrooming of the glans penis) and usually consisted of 10 2–4 s stimuli per cycle with 30 s intervals in-between cycles. The stimuli of the first cycle were just sufficient to cause mild contraction of the anal sphincter. Thereafter, with each subsequent cycle, the voltage was increased stepwise until ejaculation commenced. At this stage, the voltage was kept more or less constant but stimulus duration was increased to approximately 10 s. Ejaculates or ejaculatory fluids were usually obtained within 20 min of the first cycle and were collected in 50 ml Greiner tubes as described above.

### Semen analysis

Total volume was recorded after adding all fractions together. Ejaculatory fractions of each bull were assessed individually for the presence of spermatozoa. An aliquot of each fraction was immediately assessed for total and progressive motility and semen smears were made. Sperm concentration and percentage motile sperm were determined using automated image analysis techniques. This analysis involved a Nikon Eclipse 50i microscope (IMP, Johannesburg, South Africa) with a temperature controlled stage at 37°C (HS-50, IMP, Johannesburg, South Africa) and fitted with a Basler 312fc digital camera (Microptic SL, Barcelona, Spain). Sperm concentration and motility were assessed using a 10x negative phase objective and analysed with the Sperm Class Analyser (SCA^®^) (Microptic SL, Barcelona, Spain) version 4.1.0.0 or 5.4.0.0 at 50 frames per second. Chambered Leja® slides (Leja® slide, Leja® Products B.V., The Netherlands) with a defined volume (3 μl) and depth (20 μm) were loaded by micropipette for automated analysis. All disposables such as slides and pipette tips used were maintained at 37°C. Where sperm concentration was too high for automated analysis, the semen was diluted in Ham’s F10 culture medium (Sigma-Aldrich, Johannesburg, South Africa). At least 500 spermatozoa were analyzed for motility with SCA for each elephant. The best quality semen fraction was used to report total motility, progressive motility, sperm concentration and sperm morphology for each bull. Semen smears were processed and stained with SpermBlue® (Microptic SL, Barcelona, Spain) according to van der Horst & Maree [28] and eosin-nigrosin (Section Reproduction, Veterinary Faculty, University of Pretoria, Pretoria, S.A.) within 30 min of collection to assess sperm morphology. The eosin-nigrosin smear was made by mixing pre-heated (37°C) stain and semen on a pre-heated microscope slide at a 2:1 (stain: semen) ratio. The stained spermatozoa were evaluated for morphology using bright field microscopy at 1000x magnification. Evaluation and classification of sperm morphology of 100 spermatozoa per elephant bull was carried out according to the criteria originally described for the domestic cattle [29,30].

### Faecal sampling and androgen metabolite assay

Faecal samples were collected within 6 hours of defecation from the 11 captive bulls twice monthly and one week prior to, during and one week after each examination. Faecal matter was thoroughly mixed during collection and stored at -20°C until further processing. Frozen faecal samples were lyophilized, pulverized, and put through a nylon sieve to separate course material [31]. A weighed amount (approximately 0.05 g) of dried faecal powder was then extracted with 80% ethanol in water (3 ml) by vortex-mixing for 10–15 min and subsequent centrifugation for 5 minutes. The faecal extracts were assayed for immunoreactive androgen metabolites using an enzyme-immunoassay for 5α-Androstan-3β-ol-17-on (Epiandrosterone) which has been validated in several mammalian species [32, 33] including African and Asian elephant bulls [34, 35] for measuring faecal androgen metabolite (fAM) concentrations. Serial dilutions of faecal extracts gave displacement curves that were parallel to the respective standard curve. Sensitivity of the assay at 90% binding was 3 pg/well and the intra- and inter-assay coefficients of variation ranged between 2.4 and 11.9%. The assay was performed on microtiter plates as described by Ganswindt *et al*. (34) and conducted at the Endocrine Research Laboratory at the Faculty of Veterinary Science, University of Pretoria.

### Statistical analyses

For the ultrasound related data, descriptive statistics were calculated, histograms were plotted, and the Anderson-Darling test for normality was performed (MINITAB Statistical Software, Release 13.32, Minitab Inc, State College, PA, USA) to assess the normality assumption. Data were transformed using the natural logarithm to improve the distributional form prior to statistical analysis. The number of GnRH vaccinations was transformed into an ordinal variable for statistical modelling based on the following criteria: no GnRH vaccinations, 2 vaccinations, 3 vaccinations, 4 vaccinations, and 5 or more vaccinations. Correlations between quantitative data were assessed by calculating Spearman’s rho. The effect of multiple GnRH vaccinations was estimated within a general linear modelling approach to adjust for the repeated sampling of individual elephants. Elephant age was dichotomized based on less than 20 years of age and height was dichotomized based on the median of the data (287 cm). A random effect term was included for elephant and a first order autoregressive correlation structure was used to adjust for repeated measurements. Models included a fixed effects for number of previous GnRH vaccinations, age, height at the shoulder (used to approximate body weight by formulas described previously), and time since first vaccination. Post-hoc pairwise comparisons were adjusted using the Bonferroni method. Statistical analyses were performed in commercially available software (IBM SPSS Statistics, Version 23, International Business Machines Corp., Armonk, NY, USA) and results interpreted at the 5% level of significance. Values are presented as mean ± standard deviation (SD), unless stated otherwise.

For assessment of the faecal androgen metabolite concentration in group 1, one-way repeated measure ANOVA was applied.

## Results

In this study, it was possible to monitor 11 bulls treated with GnRH (Improvac®) over a three-year period. Another six elephants (2 wild, 4 captive) that had previously been treated with Improvac® more than eight times were examined once only (Table 1).

**Fig 2:**
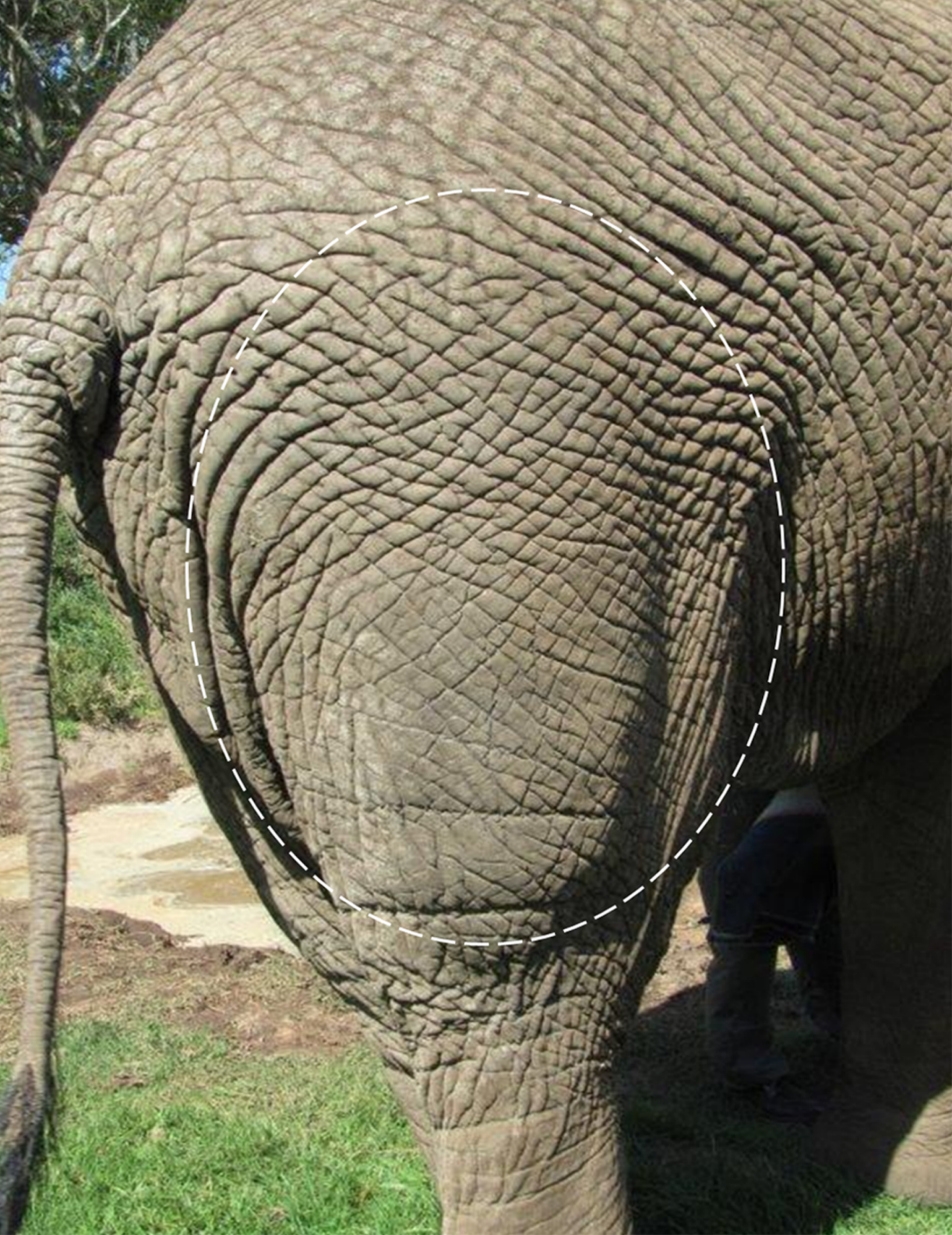
Photograph of an elephant hindleg showing swelling (circle line) one day after GnRH vaccine booster.

All bulls were healthy and in good body condition. Application of the vaccine by dart gun or hand-injection proved equally effective. Only four of the 84 (3.4%) Improvac® injections resulted in side-effects. These consisted of local swelling (Fig 2) and/or discomfort at injection site (n = 2), stiffness in hind legs (n = 1) and mild lameness which lasted one day (n = 1).

### Ultrasonographic changes of reproductive organs

#### Size of organs

Dimensions of testes, seminal vesicles, ampullae and prostate at the beginning (0–2 vaccinations) and end of the study (≥5 vaccinations) are shown in Table 2. In the seven elephants examined prior to GnRH vaccination, testis size was significantly correlated to age at the beginning of the study (r = 0.897, p = 0.0062). The mean testicular length and height of 10 bulls decreased significantly from 13.3 ± 2.6 x 15.2 ± 2.8 cm at the beginning (0–2 injections) to 7.6 ± 2.1 x 10.2 ± 1.8 cm at the end (after > 5 injections) of the study (p = 0.0009, t = 6.121, paired t-test, n = 10, Table 2). Similarly, the dimensions of the ampullae, prostate and seminal vesicles decreased significantly (Table 2).

**Table 2:** Comparative ultrasound measurements for reproductive organ area, dimesions (length x height) and circumference at start versus end of the study of group I bulls.

	Study Start	Study End	Study Start	Study End	Study Start	Study End
Organ:	Area (cm^2^)	Area (cm^2^)	Length x height^a^ (cm)	Length x height^a^ (cm)	Circumference (cm)	Circumference (cm)
**Testicle**	165.6 ± 54.6	56.5 ± 25.5	13.3 ± 2.6 x 15.2 ± 2.8	7.6 ± 2.1 x 10.2 ± 1.8	46.6 ± 9.3	29.3 ± 5.5
**Ampulla**	9.1 ± 3.0	2.2 ± 0.5	2.3 ± 0.7 x 5.1 ± 1.1	1.0 ± 0.3 x 3.2 ± 0.5	*nm*	*nm*
**Seminal** **Vesicle**	28.2 ± 13.7	14.2 ± 14.5	4.1 ± 1.6 x 9.0 ± 1.3	2.2 ± 1.4 x 7.1 ± 1.7	*nm*	*nm*
**Prostate**	13.2 ± 6.1	6.8 ± 3.3	3.6 ± 1.1 x 6.2 ± 1.2	2.1 ± 0.8 x 5.5 x 1.8	*nm*	*nm*

Results for n = 10 elephants at study start (<2 vaccinations) versus study end (≥5 vaccinations).

^a^ maximum length x height dimension of organ; *nm* = not measured

**Fig 3:**
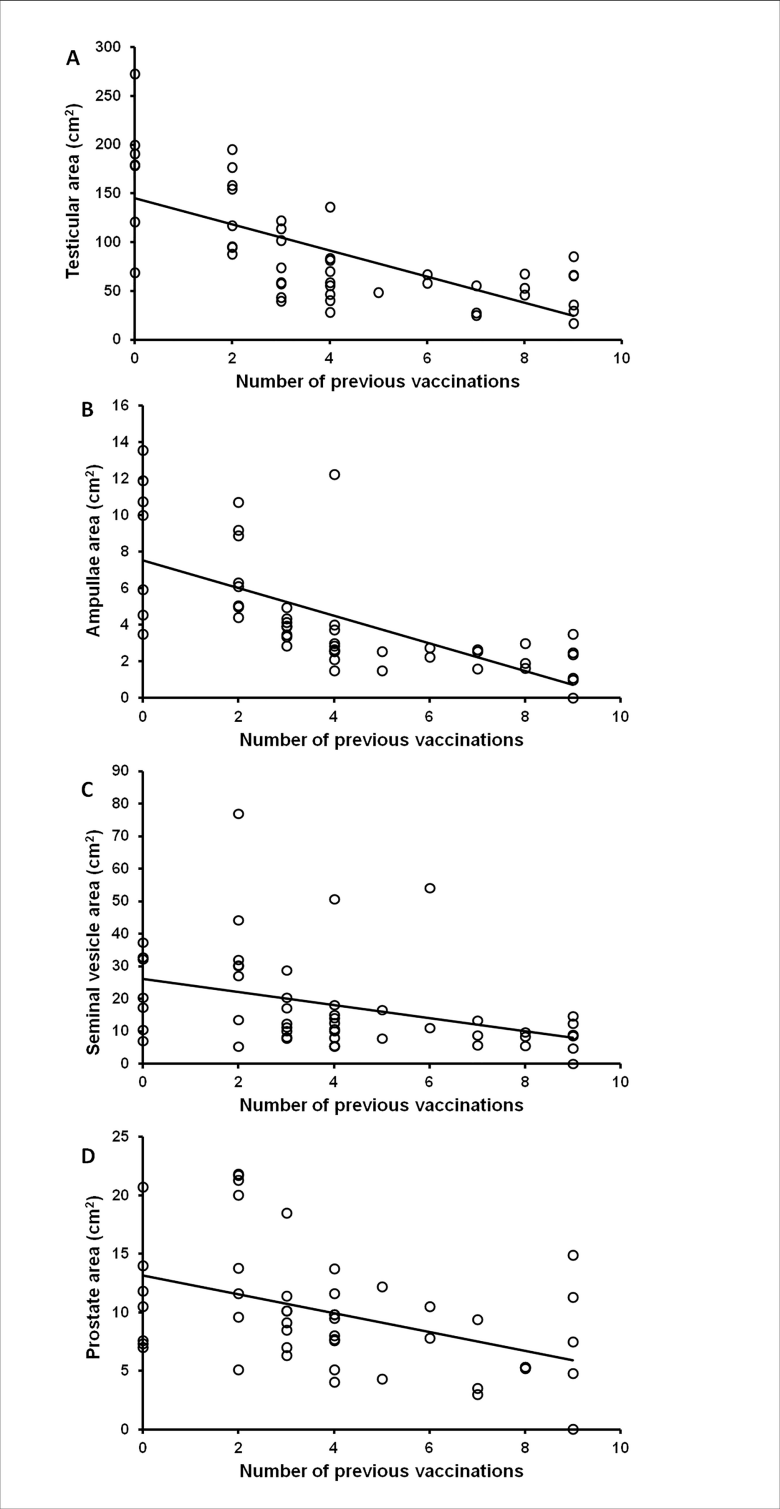
Graphs showing the negative correlation between number of GnRH vaccination and reproductive organ size in elephants bulls of group 1.

Negative correlations between age and size-adjusted organ area at the time of examination and number of vaccinations were found for the testes (Spearman’s rho = -0.728, p < 0.001, Fig 3A), ampullae (Spearman’s rho = -0.825, p < 0.001, Fig 3B), seminal vesicles (Spearman’s rho = -0.448, p = 0.001, Fig 3C) and prostate glands (Spearman’s rho = -0.436, p = 0.002, Fig 3D) (n = 17, Table 3).

**Table 3:** Univariate associations between reproductive organ size and quantitative predictor variables in 17 bull elephants.

Reproductive organ/predictor	Estimate ($\hat{\beta }$)^a^	95% CI^a^	P value
Testicular area (cm^2^)			
Age (yrs)	-0.0024	-0.0490, 0.0442	0.915
Height (cm)	-0.0016	-0.0103, 0.0072	0.716
Time since fist vaccination (days)	-0.0010	-0.0014, -0.0006	<0.001
Number of previous vaccinations	-0.1404	-0.1874, -0.0933	<0.001
Ampullae area (cm^2^)			
Age (yrs)	-0.0452	-0.0909, 0.0005	0.052
Height (cm)	-0.0095	-0.0176, -0.0013	0.025
Time since fist vaccination (days)	-0.0011	-0.0015, -0.0006	<0.001
Number of previous vaccinations	-0.1724	-0.2186, -0.1262	<0.001
Seminal vesicle area (cm^2^)			
Age (yrs)	0.0209	-0.0200, 0.0618	0.295
Height (cm)	0.0033	-0.0048, 0.0114	0.401
Time since fist vaccination (days)	-0.0008	-0.0014, -0.0002	0.009
Number of previous vaccinations	-0.1001	-0.1597, -0.0404	0.002
Prostate area (cm^2^)			
Age (yrs)	0.0202	-0.0234, 0.0637	0.333
Height (cm)	-0.0022	-0.0190, 0.0064	0.594
Time since fist vaccination (days)	-0.0009	-0.0012, -0.0006	<0.001
Number of previous vaccinations	-0.1038	-0.1462, -0.0613	<0.001

^a^ Results presented for data transformed using the natural logarithm

CI = confidence interval

The decrease in testicular and ampullar size was already significant after the second Improvac® injection. For the seminal vesicle and prostate areas, however, the differences only became significant after the third and fifth injections, respectively (Table 4). One captive bull in particular (bull # 9), responded slower compared to the other males in the study. While testis size also decreased, the size of his seminal vesicles and ampullae remained relatively unchanged throughout the study.

**Table 4:** The effect of the number of GnRH vaccinations on areas of four reproductive organs adjusted for age and size of the elephant at the time of examination. Data presented as the mean (standard error) of model predicted values.

	Number of vaccinations					
Reproductive organ size (cm^2^)	0	2	3	4	5 or more	P value*
Testes	139.4^a^^,^^b^ (1.13)	133.8^b^ (1.13)	77.4^c^ (1.13)	62.9^c^ (1.12)	44.1^d^ (1.11)	<0.001
Ampullae	7.9^a^ (1.18)	6.8^a^^,^^b^ (1.17)	3.8^b^^,^^c^ (1.17)	3.2^c^^,^^d^ (1.15)	2.1^d^ (1.12)	<0.001
Seminal vesicles	18.6^a^^,^^b^ (1.24)	21.8^a^ (1.24)	15.5^a^^,^^b^ (1.23)	12.3^a^^,^^b^ (1.21)	9.8^b^ (1.18)	0.020
Prostate glands	11.2^a^ (1.2)	13.2^a^ (1.2)	11.0^a^ (1.2)	9.0^a^ (1.19)	6.1^b^ (1.18)	<0.001

^a-d^ Means without superscripts (a-d) in common were significantly different (P < 0.05) after Bonferroni correction for multiple post-hoc tests.

*P value based on a mixed-effects linear regression model including adjustment of repeated measurements and height and age of elephant at time of sampling.

#### Ultrasonographic appearance

**Fig 4:**
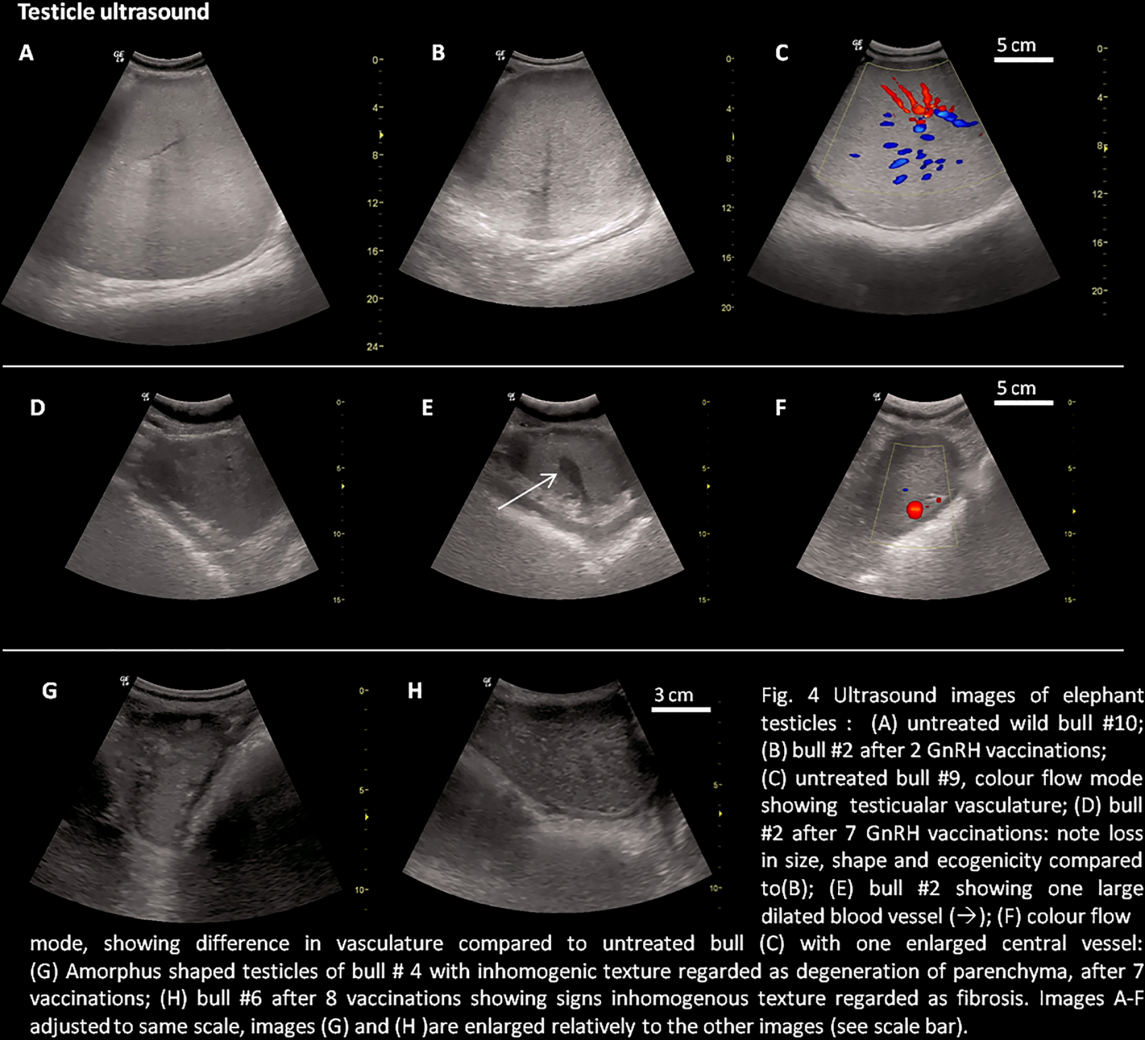
Ultrasonographic images of elephant bull testicles before and after GnRH vaccination: (A) untreated wild bull #10; (B) bull #2 after 2 GnRH vaccinations; (C) untreated bull #9, CD mode showing testicular vasculature; (D) bull #2 after 7 GnRH vaccinations: note loss in size, shape and ecogenicity compared to(B); (E) same bull #2 showing one large dilated blood vessel (→); (F) CD mode showing difference in vasculature compared to untreated bull (C) with one enlarged central vessel: (G) Amorphus shaped testicles of bull # 4 with inhomogenous texture regarded as degeneration of parenchyma, after 7 vaccinations; (H) bull #6 after 8 vaccinations showing signs inhomogenous texture regarded as fibrosis. Images A-F adjusted to same scale, images (G) and (H) are enlarged relatively to the other images (see scale bar); CD = Colour Doppler.

The testes of all bulls changed from full and round (Fig 4A–4C) at the beginning to oval and amorphous at the end of the study (Fig 4D–4H). The testicular tissue appeared to be softer and compressed by the intestines (Fig 4D–4G). The echotexture was echogenic at the beginning (Fig 4A–4C) but progressively lost echogenicity during the course of the study (Fig 4D–4G). A consistent finding in elephants treated more than five times was a dilatation of the testicular vein (Fig 4E and 4F). The colour doppler flow pattern prior to treatment was diffuse (Fig 4C) whereas after repeated treatments one central vein with less peripheral flow became evident (Fig 4F). Elephants that were treated more than five times also had small and deformed testicles (Fig 4D–4H). The appearance of the testicular tissue in six elephants that had been treated more than seven times was inhomogenouswith hyperechoic areas (Fig 4G and 4H), possibly signs of parenchymal atrophy (Fig 4G) and degeneration, resembling fibrosis (Fig 4H).

**Fig 5:**
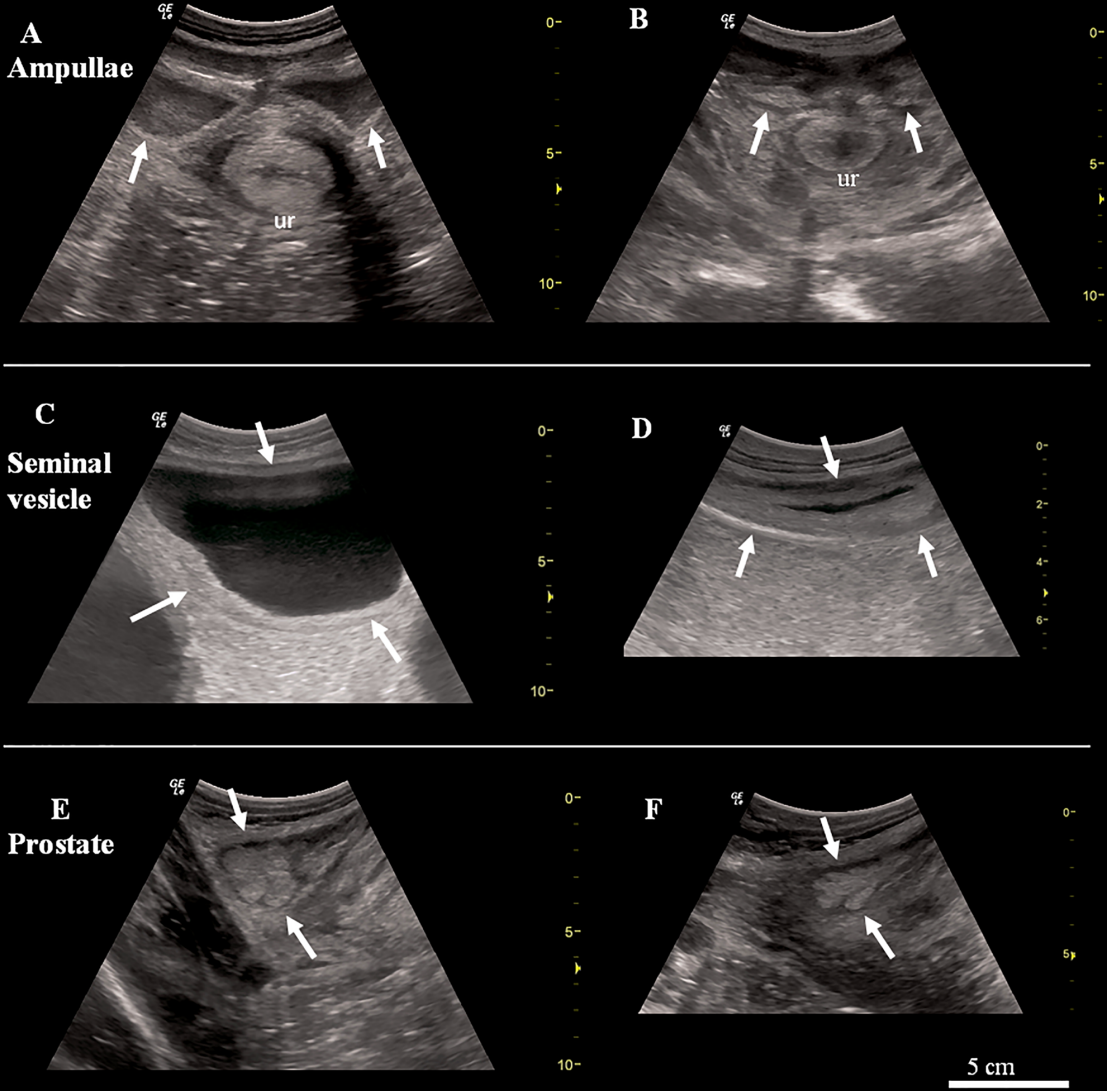
Ultrasonographic images of elephant bull accessory sex glands before and after GnRH vaccination: (A) prior to GnRH vaccination: sperm and fluid filled right and left ampullae of the ductus deferens (arrows) just before joining the urethra (ur) and (B) after 3 GnRH vaccine treatments, when ampullae yield no content anymore; (C) Large fluid filled seminal vesicles (arrows) prior to GnRH vaccination and (D) smaller size of the seminal vesicles and fluid content reduction after 7 vaccinations; (E) left prostatic lobe (arrows) after 2 vaccinations and (F) after 7 GnRH injections, note the size reduction. Image size adjusted to same scale (see scale bar).

Prior to treatment, fluid (hypoechoic) was visible in the ampullae of all mature animals (Fig 5A). The fluid content decreased over time which, after three vaccinations, significantly reduced the size of the ampullae. After five vaccinations, fluid was no longer visible in the ampullae (Fig 5B). The fluid within the seminal vesicles was clearly visible in most bulls at the beginning of the study (Fig 5C) and only appeared to be affected after more than four vaccinations. By the end of the study, seminal vesicle fluid had disappeared completely in seven bulls of group 1. In the remaining animals, small anechoic fluid filling was visible (Fig 5D and 5F). The size of the prostate also decreased (Fig 5E, pre-treatment); the effect becoming clearly visible after more than five vaccinations (Fig 5F; Table 4).

### Semen collection and evaluation

Penis extrusion of elephants in standing sedation was sometimes spontaneous and probably as a result of the sedative drugs. If not, extrusion occurred during the cleaning of the rectum and trans-rectal massaging. In most cases, continuous trans-rectal massaging of the underlying organs resulted in the development of a fully extended penis. In two cases, urine dribbling was observed prior to massaging. When the urethra was stimulated in the correct area, however, urine dribbling ceased and a semen sample was obtained. Urine contamination of some fractions was recorded in four of 31 manual collections. One (bull # 5) of the seven males assessed prior to treatment was 8 years old and, according to the appearance of reproductive organs, low testosterone levels and absence of spermatozoa in the ejaculate, was assessed as prepubertal. This male was therefore not incorporated in the semen evaluation over time.

In untreated males, transrectal massage prior to the first vaccination produced ejaculate volumes of 8–50 ml. Electro-ejaculation of the immobilised naïve wild bulls was highly successful and produced ejaculate volumes of 43 ml (bull #11) and 175 ml (bull #10) prior to vaccination. Electro-ejaculation of wild bulls on longstanding treatment (group 2; bulls #14 and #15) produced only drops of sperm-free seminal plasma.

**Fig 6:**
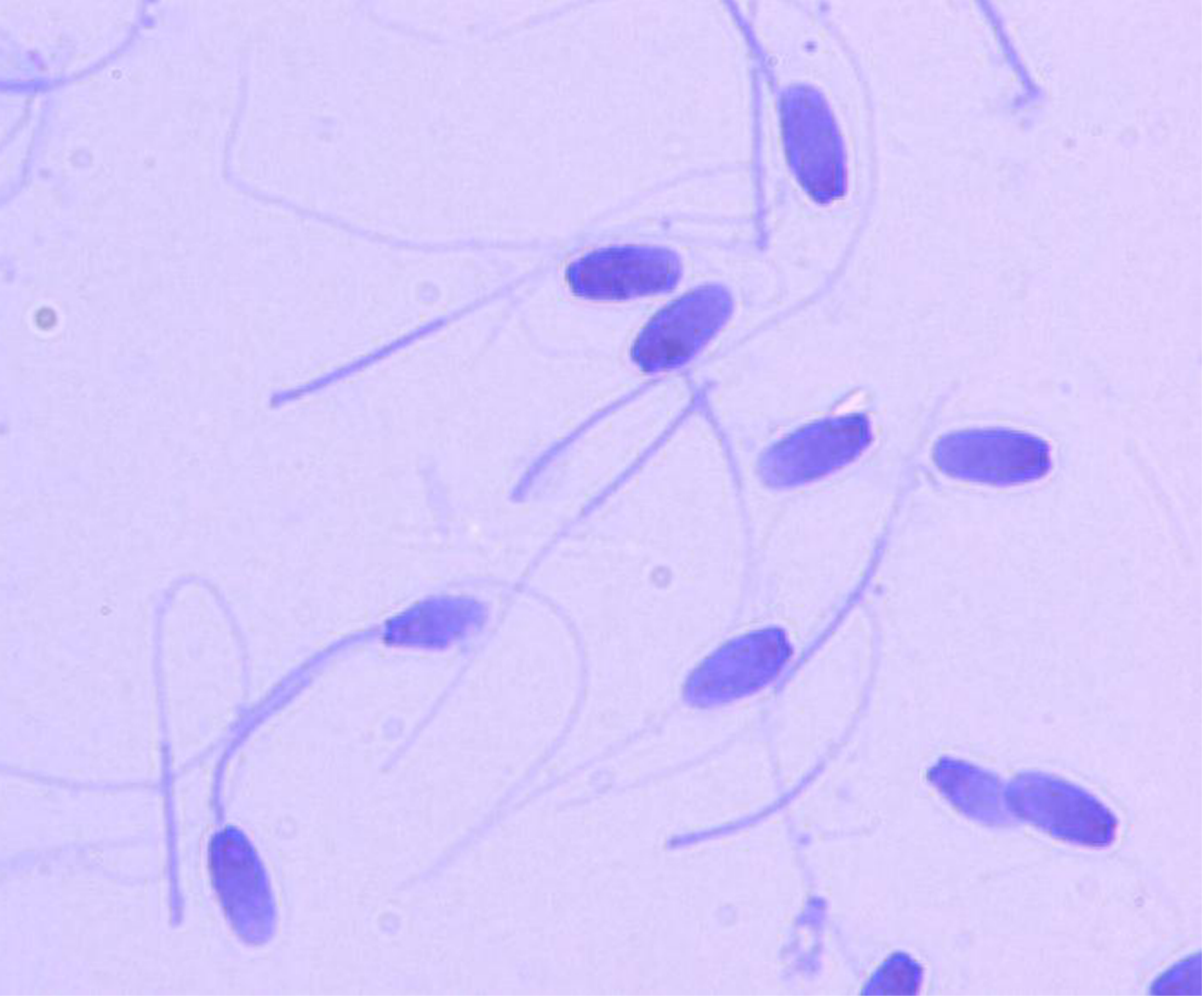
Microscopic image of stained elephant spermatozoa 8.5 months after the first GnRH vaccination (= 3 injections: Primer, 1^st^ and 2^nd^ booster), showing the example of tail and head separation, stain: SpermBlue®, magnification: x1000.

Table 5 summarizes the semen quality of bulls prior to treatment and after two, three, four to five and more than eight vaccinations. In bulls examined prior to treatment (n = 6), ejaculate volume, sperm concentration, sperm motility and percentage normal spermatozoa were recorded (Table 5). The median values of each these variables decreased with increasing numbers of vaccine treatments, with no spermatozoa present in ejaculates from bulls given more than eight vaccinations (Table 5). However, there were strong individual differences. For example, considerable amounts of ejaculatory fluids were still produced in some bulls, containing a substantial concentration of spermatozoa even after 4vaccinations. The percentage normal spermatozoa decreased significantly from 33.8–93.8-% prior to vaccination to only 0.7–26% after three treatments (six months after the primary vaccination). No spermatozoa with normal morphology were recorded for all bulls after four or more vaccinations, when only few or no spermatozoa were present in the ejaculates. Only one bull (bull #9) had a sperm count of 220x10^6^/ml at this stage. Here, however, no sperm motility was recorded and 43% detached heads were noted. A consistent finding in all bulls was head-tail separation, which involved as much as 53–85% of spermatozoa after three vaccinations (Fig 6). All bulls examined were azoospermic after more than eight treatments and only small amounts of sperm-free seminal fluid were collected.

**Table 5:** Effect of number of GnRH vaccinations with Improvac® on ejaculate variables observed over the course of the study in African elephant bulls of group 1.

***Number of vaccinations*:**	***Ejaculate volume (ml)***	***Sperm conc*. *(x10***^***6***^ ***/ml)***	***Total sperm motility (%)***	***Morphology (% normal sperm)***
**untreated (n = 6)**				
**MEDIAN (range)**	**32.8** (8–175)	**1172.5** (410–4000)	**62.5** (0–90)	**57.6** (33.8–93.8)
**2 vaccinations (n = 6)**				
**MEDIAN (range)**	**13.8** (7–169.5)	**445** (47–1540)	**0** (0–68)	**53.9** (33.3–78.7)
**3 vaccinations (n = 7)**				
**MEDIAN (range)**	**5.0** (0.5–29)	**294** (0–9800)	**0** (0–2)	**6** (0.7–26)
**4–5 vaccinations (n = 8)**				
**MEDIAN (range)**	**17.5** (0–65)	**0.05** (0–220)	0	0
**> 8 vaccinations (n = 6)**				
**MEDIAN (range)**	**0.2** (0–27.8)	0	0	0

### Faecal androgen metabolite concentrations

**Fig 7:**
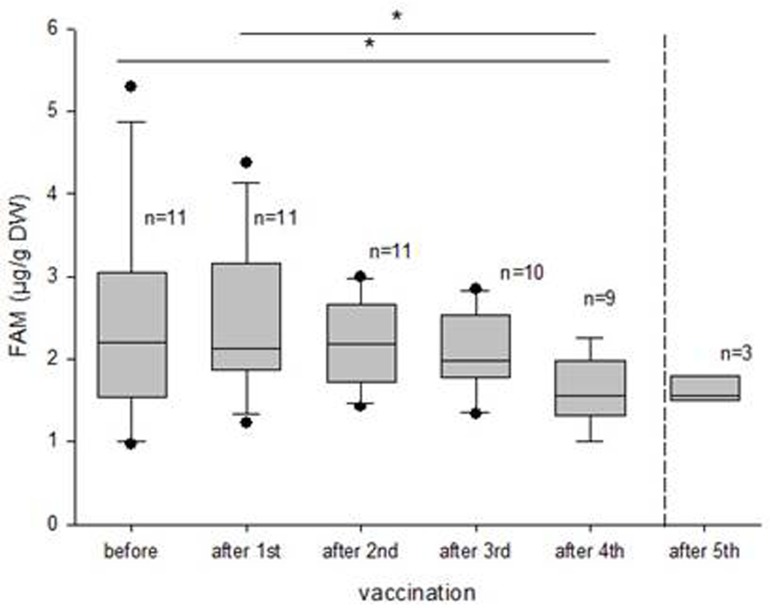
Boxplots depicting the decline in fecal androgen metabolite (fAM) concentration after numerous GnRH vaccinations in elephant bulls of group 1. After the fourth injection, a significant reduction in fAM was noticed.

In group 1, median fAM concentrations in 11 captive bulls were predominantly low at the start of the study, ranging from 0.96–5.29 μμg/g DW, depicting comparable non-musth levels (overall median: 2.19 μg/g DW). The high variability in individual fAM level detected between untreated bulls can probably be attributed to the different age classes and social ranks. A significant reduction in fAM concentrations was seen after four vaccinations (one year after the primary immunization, Fig 7) with an overall median fAM concentration decreasing by 29% to 1.56 μg/g DW (range: 0.99–2.26 μg/g DW). Concurrently, during the course of the study, none of these captive males showed any signs of musth.

## Discussion

The current study in African elephant bulls showed that repeated injections of the GnRH vaccine Improvac® had significant effects on the reproductive organs, semen quality, fAM concentrations, and the suppression of musth.

### Effects on androgen concentrations

The current study clearly demonstrated the suppressive impact of the GnRH vaccine on the HPG-axis and finally on the Leydig cell function, resulting in decreased fAM concentrations and thus confirming the findings of previous studies in African [13, 14, 17] and Asian elephant bulls [12, 15]. The fAM concentrations were significantly reduced and reached baseline concentrations, similar to post-musth concentrations reported for adult wild bulls in the Kruger National Park [31], in all bulls of group 1 after four or more vaccinations. In a study on Asian elephant bulls, a rise in antibody titres was seen after three monthly vaccinations with Improvac® (600–1200 μg). The antibody titre was negatively correlated with testosterone concentrations [12].

Our findings further indicate that treatment intervals of 5–7 months following the first booster, delivered six weeks after the primary vaccination, are sufficient to control androgen secretion and prevent the state of musth during the treatment period. Previous studies also showed that after a good initial immunization protocol, longer vaccination intervals of 5–7 months are effective in suppressing testosterone levels and musth [12, 13, 36].

By suppressing testosterone, GnRH vaccination could potentially be applied to mitigate problem elephant cases. Wild African elephant bulls are reportedly more likely to raid crops [37] and break fences [38] compared to females. Furthermore, the GnRH vaccination could have several benefits for captive elephant bulls and their handlers in zoos and elephant sanctuaries including: i) suppression of testosterone driven aggressive behaviours and musth; ii) avoiding animal welfare problems such as prolonged separation and chaining during the musth phase; and iii) facilitating formation of bachelor herds in zoos or multi-male constellations within female groups.

In captive breeding populations in Europe and North America, more male than female elephant calves are born [39]. The housing and management of these excess males as they mature is a rapidly looming problem. Keeping males that are regularly treated with GnRH vaccine in stable bachelor groups may be a possible management solution for surplus males. Alternatively, if pre-pubertal vaccination is applied, males may remain part of the female group as already described in a single Asian elephant male [15] and observed in the wild 15 year-old male (bull # 11). In the age group 12–16 years, testosterone output starts to increase and this is particularly problematic in captive young bulls in South Africa (Bertschinger, personal communication). This is also the age when young bulls in the wild reach puberty and leave their natal herds [40, 41]. Currently, bulls in musth are hobbled and short leg chains are applied to restrict movement for extended periods. Access to water and food is restricted and tranquillizers may even be administered [42]. All these measures present significant welfare issues. Efforts to suppress musth with GnRH analogues such as leuprolide acetate [43] and deslorelin implants [44] were largely unsuccessful. The advantage of GnRH vaccines is that they have an immunological action and do not rely on intervention with exogenous hormones. Furthermore, they are more cost effective than GnRH analogues, fewer treatments are required and they can be delivered remotely with a dart gun. While the vaccine cannot change learned behaviours or individual personalities, it may mitigate testosterone driven traits particularly if applied at a pre-pubertal age.

### Effects on reproductive organs and semen quality

We found a significant decrease in testis size as well as all accessory sex glands after multiple treatments with the GnRH vaccine. The accessory sex glands, such as seminal vesicles, ampullae of the ductus deferens and prostate were shown to be reliable markers for predicting breeding potential in male elephants [22, 23]. Gonadal atrophy has been described in several GnRH vaccine studies in various species [5]. The effect of GnRH vaccination on testis size was considerable with a mean reduction in diameter of 60% in our study. This is consistent with a 70%, 40–80% and about 50% reduction in testicular weight of pigs [45], ram lambs [46] and domestic bulls [47] treated with GnRH vaccines, respectively.

Not surprising was the effect of GnRH vaccine treatment on size and fluid content of the ampulla and prostate of bulls in the current study. The accessory sex glands are androgen-dependent for normal function. GnRH analogues, which also down-regulate FSH and LH release, decrease prostate size and are thus commonly used for treatment of benign prostate hypertrophy in dogs [48] and humans [49], and prostate cancer cancer in humans [50]. The ampullae of the vas deferens offer temporary sperm storage in elephant bulls [23]. A reduction in spermatogenesis would thus lead to less luminal content in this paired organ. The effect on seminal vesicles was also visible, however, androgen deprivation appears to have had less effect on their fluid content.

From a contraception point of view, the most important effect of the GnRH vaccine Improvac® was the one on semen quality. Sperm production was altered after three GnRH immunisations, and spermatozoa were absent (azoospermia) after more than four vaccination in 50% of elephant bulls. The remaining bulls had only few abnormal and immotile spermatozoa present in their ejaculates. All these findings on sperm alteration are consistent with those in mature stallions immunized with a GnRH vaccine [51]. Breeding potential would seem to be markedly affected after the first three injections (six months) and most elephant bulls could be expected to be infertile after 4–5 injections (1–1.5 year of treatment). The arrest of spermatogenesis may not be consistent in all bulls, clearly demonstrated by elephant #9. The accessory sex glands of this bull were also less affected than the other bulls. Individual responses should be considered, and the ability to produce viable spermatozoa may be retained for prolonged periods. Nevertheless, the sperm motility of bull #9 decreased from 35% prior to treatment to 0% after six months, morphology from 34% to 1% after 12 months and only isolated sperm were observed after 36 months. Azoospermia was seen in all elephant bulls treated for more than five years and 4/8 bulls treated for three years. An effect of age on response to GnRH vaccine could not be confirmed for the elephants in our study.

### Side effects and reversibility

Although other studies did not report side effects in elephants treated with GnRH vaccines [15–18], we noticed a few negative reactions in a small percentage of vaccinations. These temporary side effects were seen as local swelling (Fig 2), stiffness and mild lameness. When Asian elephants were vaccinated into the neck muscle region, no problems were reported [12, 15]. However, Bertschinger and Sills [13] noted occasional stiffness and swellings in African elephant bulls injected into the neck muscles. The adjuvant used in Improvac® is a diphtheria toxoid and animals tend to show greater reactions if it is administered subcutaneously or into intermuscular fascia. In mares and stallions, minor side effects are seldom seen when Improvac® is administered by deep intramuscular injection into the gluteal muscle mass [8]. At the same time, another GnRH vaccine and adjuvant administered to White-tailed deer (*Cervus dama*) resulted in abscess and/or granuloma formation in nearly all animals after the first immunization [52]. Thus, site of injection should be selected according to experiences in different species. Larger volumes should be administered in two injection sites. Care should be taken to ensure deep intramuscular injection to reduce side effects depending on the adjuvant used.

A very important question, particularly for some species or circumstances, is the reversibility of GnRH vaccines. We did not plan to test reversibility of the GnRH vaccine in the current study, but it certainly needs to be investigated. The loss of testosterone at a young age may lead to retarded development of the penis, testicles and accessory sex glands in elephants [13, 5]. In a previous study, treatment of a young male Asian elephant discontinued after being immunised every 5–12 months for a period of more than six years. It has now been four years since the last booster and his testosterone concentration remains at baseline (personnel communication Charlie Gray, African Lion Safari, Canada, 19.07.2016).

An alerting finding was the ultrasonographic appearance of the testes following a number of GnRH vaccinations (Fig 4B–4E). The loss of echogenicity and testicular architecture, and some of the sperm defects (Fig FB-E) observed, are possibly signs of testicular degeneration. The observations were also made in the mature bulls and indicate that permanent damage may follow consecutive treatments as early as after 1.5 years. Severe testicular pathologies such as seminiferous tubule degeneration, segmental tubular aspermatogenesis, decreased cross-sectional diameter of seminiferous tubules, decreased cytoplasmic volume of Leydig cells, interstitial fibrosis, and total aspermatogenesis have been observed in White-tailed deer treated three times with a GnRH vaccine [52]. Although testicular histology has not been performed on GnRH vaccine-treated elephant bulls, some of our ultrasonographic observations indicate similar processes could have occurred (Fig 4B–4E). It was not possible to objectively assess bull behaviour before and at intervals after GnRH vaccine treatment. However, it should be noted that before commencement of treatment, two of the captive bulls were highly aggressive towards their handlers and one wild bull was in full musth. The aggressive behaviour and musth ceased after two immunisations and did not recur by the end of the study. None of the other bulls showed sexual interest, musth or musth related behaviour throughout the study, despite many being at an age where musth occurs regularly or intermittently. There was no noticeable change to the masculine appearance of the males in our study, likely because the period of observation was relatively short. Even bull #17, who was 34 years at the time of examination and had been on treatment for 13 years, displayed normal masculine appearance, body growth rate and tusk growth.

In summary, the complete absence of spermatozoa in ejaculates of long-term treated bulls, baseline fAM concentrations and a lack of sexual interest suggest a castration-like effect as a result of GnRH vaccination.

Although our initial results show an impressive effect already within 1.5 years of vaccination with Improvac®, more research is needed to determine optimal treatment intervals, reversibility of the treatment, and effects in juvenile versus adult African elephant bulls. Furthermore, the effects on social, reproductive and hierarchical behaviours, possible demographic impacts and effects on bone metabolism need to be investigated.

## Conclusions

The GnRH vaccine Improvac®, originally developed for the pig industry, appears to have a potent effect on African elephant bulls. Regular boosters at 5–7 month intervals are sufficient to down-regulate testicular size, testosterone levels and spermatogenesis in male elephants. If elephants are treated continuously for more than 2–3 years (4–8 times), irreversible effects are possible. For the use as a contraceptive measure in wild elephant bulls, single-shot, long-acting vaccines would be more practical. On the other hand this may affect reversibility.

## References

[1] Millar RP. GnRHs and GnRH receptors. Anim Reprod Sci. 2005;88(1–2):5–28. doi: 10.1016/j.anireprosci.2005.05.032 16140177 10.1016/j.anireprosci.2005.05.032

[2] Kirkpatrick JF, Lyda RO, Frank KM. Contraceptive vaccines for wildlife: a review. Am J Reprod Immunol. 2011;66(1):40–50. doi: 10.1111/j.1600-0897.2011.01003.x 21501279 10.1111/j.1600-0897.2011.01003.x

[3] Dowsett K, Knott L, Tshewang U, Jackson A, Bodero D, Trigg T. Suppression of testicular function using two dose rates of a reversible water soluble gonadotrophin releasing hormone (GnRH) vaccine in colts. Aust Vet J. 1996;74(3):228–35. 8894040 10.1111/j.1751-0813.1996.tb15410.x

[4] Janett F, Stump R, Burger D, Thun R. Suppression of testicular function and sexual behavior by vaccination against GnRH (Equity™) in the adult stallion. Anim Reprod Sci. 2009;115(1):88–102. 19128902 10.1016/j.anireprosci.2008.11.011

[5] Ferro V, Khan M, McAdam D, Colston A, Aughey E, Mullen A, et al Efficacy of an anti-fertility vaccine based on mammalian gonadotrophin releasing hormone (GnRH-I)—a histological comparison in male animals. Vet Immunol Immunopathol. 2004;101(1):73–86. 15261694 10.1016/j.vetimm.2004.03.011

[6] Karaconji B, Lloyd B, Campbell N, Meaney D, Ahern T. Effect of an anti‐gonadotropin‐releasing factor vaccine on sexual and aggressive behaviour in male pigs during the finishing period under Australian field conditions. Aust Vet J. 2015;93(4):121–3. doi: 10.1111/avj.12307 25817977 10.1111/avj.12307

[7] Balet L, Janett F, Hüsler J, Piechotta M, Howard R, Amatayakul-Chantler S, et al Immunization against gonadotropin-releasing hormone in dairy cattle: Antibody titers, ovarian function, hormonal levels, and reversibility. J Dairy Sci. 2014;97(4):2193–203. doi: 10.3168/jds.2013-7602 24565325 10.3168/jds.2013-7602

[8] Botha A, Schulman M, Bertschinger H, Guthrie A, Annandale C, Hughes S. The use of a GnRH vaccine to suppress mare ovarian activity in a large group of mares under field conditions. Wildlife Res. 2008;35(6):548–54.

[9] Miller LA, Johns BE, Killian GJ. Immunocontraception of White‐Tailed Deer with GnRH Vaccine. Am J Reprod Immunol. 2000;44(5):266–74. 11125787 10.1111/j.8755-8920.2000.440503.x

[10] Miller LA, Rhyan JC, Drew M. Contraception of bison by GnRH vaccine: a possible means of decreasing transmission of brucellosis in bison. J Wildlife Dis. 2004;40(4):725–30. 10.7589/0090-3558-40.4.725 15650090

[11] Quy RJ, Massei G, Lambert MS, Coats J, Miller LA, Cowan DP. Effects of a GnRH vaccine on the movement and activity of free-living wild boar (Sus scrofa). Wildlife Res. 2014;41(3):185–93.

[12] Somgird C, Homkong P, Sripiboon S, Brown JL, Stout TA, Colenbrander B, et al Potential of a gonadotropin-releasing hormone vaccine to suppress musth in captive male Asian elephants (Elephas maximus). Anim Reprod Sci. 2016;164:111–20. doi: 10.1016/j.anireprosci.2015.11.019 26656504 10.1016/j.anireprosci.2015.11.019

[13] Bertschinger HJ, Sills ES. Contraceptive applications of GnRH-analogs and vaccines for wildlife mammals of southern Africa: current experience and challenges Sills S, editor. New York: Nova Science Publications Inc; 2013.

[14] De Nys HM, Bertschinger HJ, Turkstra J, Colenbrander B, Palme R, Human A. Vaccination against GnRH may suppress aggressive behaviour and musth in African elephant (Loxodonta africana) bulls: a pilot study. J S Afr Vet Assoc. 2010;81(1):8–15. 20649148 10.4102/jsava.v81i1.88

[15] Lueders I, Hildebrandt TB, Gray C, Botha S, Rich P, Niemuller C. Supression of testicular function in a male Asian elephant (Elephas maximus) treated with gonadotropin-releasing hormone vaccines. J Zoo Wildl Med. 2014;45(3):611–9. doi: 10.1638/2013-0233R.1 25314829 10.1638/2013-0233R.1

[16] Boedeker NC, Hayek L.-A. C., Murray S., de Avila D. M., and Brown J. L. Effect of a gonadotropin-releasing hormone vaccine on ovarian cyclicity and uterine morphology of an Asian elephant (Elephas maximus). J Zoo Wildlife Med. 2012;43:603–14. 10.1638/2011-0270.1 23082526

[17] Somgird C, Homkong P, Sripiboon S, Brown JL, Stout TA, Colenbrander B, et al Potential of a gonadotropin-releasing hormone vaccine to suppress musth in captive male Asian elephants (Elephas maximus). Anim Reprod Sci. 2015;164:111–20. doi: 10.1016/j.anireprosci.2015.11.019 26656504 10.1016/j.anireprosci.2015.11.019

[18] Marais HJ, Hendrickson DA, Stetter M, Zuba JR, Penning M, Siegal-Willott J, et al Laparoscopic vasectomy in African savannah elephant (Loxodonta africana); surgical technique and results. J Zoo Wildlife Med. 2013;44(4s):S18–S20. 10.1638/1042-7260-44.4S.S18 24437080

[19] Laws RM, Parker IS, Johnstone RC. Elephants and their habitats. The ecology of elephants in North Bunyoro Uganda: Clarendon Press; 1975.

[20] Johnson OW, Buss IO. Molariform teeth of male African elephants in relation to age, body dimensions, and growth. J Mammal. 1965;46(3):373–84. 14343911

[21] Lueders I, Tindall B, Young D, van der Horst G, Botha S, Luther I, et al Standing sedation with medetomidine and butorphanol in captive African elephants (Loxodonta africana). Vet J. 2016;209:190–2. doi: 10.1016/j.tvjl.2015.07.014 26831175 10.1016/j.tvjl.2015.07.014

[22] Hildebrandt TB, Göritz F, Pratt NC, Schmitt DL, Quandt S, Raath J, et al Reproductive assessment of male elephants (Loxodonta africana and Elephas maximus) by ultrasonography. J Zoo Wildlife Med. 1998;29(2):114–28. 9732024

[23] Hildebrandt TB, Hermes R, Pratt NC, Fritsch G, Blottner S, Schmitt DL, et al Ultrasonography of the urogenital tract in elephants (Loxodonta africana and Elephas maximus): an important tool for assessing male reproductive function. Zoo Biol. 2000;19(5):333–45.

[24] Schmitt DL, Hildebrandt TB. Manual collection and characterization of semen from Asian elephants (Elephas maximus). Anim Reprod Sci. 1998;53(1–4):309–14. 9835384 10.1016/s0378-4320(98)00120-1

[25] Portas T, Bryant B, Göritz F, Hermes R, Keeley T, Evans G, et al Semen collection in an Asian elephant (Elephas maximus) under combined physical and chemical restraint. Aust Vet J. 2007;85(10):425–7. doi: 10.1111/j.1751-0813.2007.00207.x 17903132 10.1111/j.1751-0813.2007.00207.x

[26] Howard J, Bush M, De Vos V, Wildt D. Electroejaculation, semen characteristics and serum testosterone concentrations of free-ranging African elephants (Loxodonta africana). J Reprod Fertil. 1984;72(1):187–95. 6471047 10.1530/jrf.0.0720187

[27] Hermes R, Saragusty J, Göritz F, Bartels P, Potier R, Baker B, et al Freezing African elephant semen as a new population management tool. PloS one. 2013;8(3):e57616 doi: 10.1371/journal.pone.0057616 23483917 10.1371/journal.pone.0057616 PMC3590205

[28] Van der Horst G, Maree L. SpermBlue®: A new universal stain for human and animal sperm which is also amenable to automated sperm morphology analysis. Biotech Histochem. 2009;84(6):299–308. doi: 10.3109/10520290902984274 19488904 10.3109/10520290902984274

[29] Campbell R, Dott H, Glover T. Nigrosin eosin as a stain for differentiating live and dead spermatozoa. J Agr Sci. 1956;48(01):1–8.

[30] Barth AD, Oko R. Abnormal morphology of bovine spermatozoa. Ames: Iowa State University Press; 1989.

[31] Ganswindt A, Muenscher S, Henley M, Henley S, Heistermann M, Palme R, et al Endocrine correlates of musth and the impact of ecological and social factors in free-ranging African elephants (Loxodonta africana). Horm Behav. 2010;57(4):506–14. 20188104 10.1016/j.yhbeh.2010.02.009

[32] Möhle U, Heistermann M, Palme R, Hodges J. Characterization of urinary and fecal metabolites of testosterone and their measurement for assessing gonadal endocrine function in male nonhuman primates. Gen Comp Endocr. 2002;129(3):135–45. 12460597 10.1016/s0016-6480(02)00525-7

[33] Palme R, Möstl E. Biotin-streptavidin enzyme immunoassay for the determination of oestrogens and androgens in boar faeces In: Görög S, editor. Advances of Steroid Analysis. Budapest: Akadémiai Kiadó; 1994 p. 111–7.

[34] Ganswindt A, Heistermann M, Borragan S, Hodges JK. Assessment of testicular endocrine function in captive African elephants by measurement of urinary and fecal androgens, Zoo Biology, 2002;21(1): 27–36.

[35] Ghosal R, Ganswindt A, Seshagiri PB, Sukumar R. Endocrine correlates of musth in free-ranging Asian elephants (*Elephas maximus*) determined by non-invasive faecal steroid hormone metabolite measurements. PloS One. 2013;8(12):e84787 doi: 10.1371/journal.pone.0084787 24358371 10.1371/journal.pone.0084787 PMC3866162

[36] Rajapaska RC, I.P.G.H.U. Dissanayaka, C.Somgird, C. Thitaram, A. Sirimalaisuwan, P.G.A. Pushpakumara, B. Colenbrander, J.L. Brown, B.M.A.O. Perera, T.A.E. Stout, editor Efficacy of GnRH vaccination for suppressing musth and aggressive behavior in male Asian elephants. EU-Asia Link Project Symposium “Managing the Health and Reproduction of Elephant Populations in Asia”; 2010; Faculty of Veterinary Medicine, University Chiang May, Thailand. Chiang Mai, Thailand: Nuntapun Printing.

[37] Chiyo PI, Moss CJ, Alberts SC. The influence of life history milestones and association networks on crop-raiding behavior in male African elephants. PloS One. 2012;7(2):e31382 doi: 10.1371/journal.pone.0031382 22347468 10.1371/journal.pone.0031382 PMC3275604

[38] Mutinda M, Chenge G, Gakuya F, Otiende M, Omondi P, Kasiki S, et al Detusking fence-breaker elephants as an approach in human-elephant conflict mitigation. PloS One. 2014;9(3):e91749 doi: 10.1371/journal.pone.0091749 24614538

[39] Saragusty J, Hermes R, Goritz F, Schmitt DL, Hildebrandt TB. Skewed birth sex ratio and premature mortality in elephants. Anim Reprod Sci. 2009;115(1–4):247–54. doi: 10.1016/j.anireprosci.2008.10.019 19058933 10.1016/j.anireprosci.2008.10.019

[40] Poole J H. Sex differences in the behavior of African elephants In: Short R.V. & Balaban E. (eds) The differences between the sexes. Cambridge University Press, Cambridge, 1994;331–346.

[41] Bertschinger, Henk, Delsink Audrey, van Altena JJ, Kirkpatrick Jay, Killian Hanno, Ganswindt Andre, Slotow Rob, Castley Guy. Chapter 6: Reproductive control of elephants. In: Elephant Management: A Scientific Assessment for South Africa. Eds RJ Scholes and KG Mennel, 2008;257–328.

[42] Lincoln G, Ratnasooriya W. Testosterone secretion, musth behaviour and social dominance in captive male Asian elephants living near the equator. J Reprod Fertil. 1996;108(1):107–13. 8958836 10.1530/jrf.0.1080107

[43] de Oliveira CA, West GD, Houck R, Leblanc M. Control of musth in an Asian elephant bull (Elephas maximus) using leuprolide acetate. J Zoo Wildlife Med. 2004;35(1):70–6. 10.1638/02-091 15193077

[44] Brown J, Bush M, Wildt D, Raath J, de Vos V, Howard J. Effects of GnRH analogues on pituitary–testicular function in free-ranging African elephants (Loxodonta africana). J Reprod Fertil. 1993;99(2):627–34. 8107048 10.1530/jrf.0.0990627

[45] Jaros P, Bürgi E, Stärk K, Claus R, Hennessy D, Thun R. Effect of active immunization against GnRH on androstenone concentration, growth performance and carcass quality in intact male pigs. Livest Prod Sci. 2005;92(1):31–8.

[46] Kiyma Z, Adams T, Hess B, Riley M, Murdoch W, Moss G. Gonadal function, sexual behavior, feedlot performance, and carcass traits of ram lambs actively immunized against GnRH. J Anim Sci. 2000;78(9):2237–43. 10985393 10.2527/2000.7892237x

[47] Cook R, Popp J, Kastelic J, Robbins S, Harland R. The effects of active immunization against GnRH on testicular development, feedlot performance, and carcass characteristics of beef bulls. J Anim Sci. 2000;78(11):2778–83. 11063298 10.2527/2000.78112778x

[48] Gobello C. Dopamine agonists, anti-progestins, anti-androgens, long-term-release GnRH agonists and anti-estrogens in canine reproduction: A review. Theriogenology. 2006;66(6):1560–7. 16542717 10.1016/j.theriogenology.2006.02.005

[49] Gabrilove J, Levine A, Kirschenbaum A, Droller M. Effect of a GnRH analogue (leuprolide) on benign prostatic hypertrophy. J Clin Endocr Metab. 1987;64(6):1331–3. doi: 10.1210/jcem-64-6-1331 2437142 10.1210/jcem-64-6-1331

[50] Simms MS, Scholfield DP, Jacobs E, Michaeli D, Broome P, Humphreys JE, et al Anti-GnRH antibodies can induce castrate levels of testosterone in patients with advanced prostate cancer. Brit J Cancer. 2000;83(4):443 doi: 10.1054/bjoc.2000.1315 10945488 10.1054/bjoc.2000.1315 PMC2374644

[51] Malmgren L, Andresen Ø, Dalin AM. Effect of GnRH immunization on hormonal levels, sexual behaviour, semen quality and testicular morphology in mature stallions. Equine Vet J. 2001;33:75–83. 11191615 10.2746/042516401776767340

[52] Curtis PD, Richmond ME, Miller LA, Quimby FW. Physiological effects of gonadotropin-releasing hormone immunocontraception on white-tailed deer. Human-Wildlife Conflicts. 2008;2(1):68–79.

